# Effects of Delivering Guanidinoacetic Acid or Its Prodrug to the Neural Tissue: Possible Relevance for Creatine Transporter Deficiency

**DOI:** 10.3390/brainsci12010085

**Published:** 2022-01-07

**Authors:** Enrico Adriano, Annalisa Salis, Gianluca Damonte, Enrico Millo, Maurizio Balestrino

**Affiliations:** 1 Department of Neuroscience, Ophthalmology, Genetics, Maternal-Infantile Sciences, University of Genova, Largo Paolo Daneo 3, 16132 Genova, Italy; adriano@neurologia.unige.it; 2 Department of Experimental Medicine, Section of Biochemistry, University of Genova, Viale Benedetto XV 1, 16132 Genova, Italy; Annalisa.Salis@unige.it (A.S.); gianluca.damonte@unige.it (G.D.); enrico.millo@unige.it (E.M.); 3 IRCCS Ospedale Policlinico San Martino, 16132 Genova, Italy

**Keywords:** creatine, transporter, guanidinoacetic acid, creatine transporter deficiency, hereditary

## Abstract

The creatine precursor guanidinoacetate (GAA) was used as a dietary supplement in humans with no adverse events. Nevertheless, it has been suggested that GAA is epileptogenic or toxic to the nervous system. However, increased GAA content in rodents affected by guanidinoacetate methyltransferase (GAMT) deficiency might be responsible for their spared muscle function. Given these conflicting data, and lacking experimental evidence, we investigated whether GAA affected synaptic transmission in brain hippocampal slices. Incubation with 11.5 μM GAA (the highest concentration in the cerebrospinal fluid of GAMT-deficient patients) did not change the postsynaptic compound action potential. Even 1 or 2 mM had no effect, while 4 mM caused a reversible decrease in the potential. Guanidinoacetate increased creatine and phosphocreatine, but not after blocking the creatine transporter (also used by GAA). In an attempt to allow the brain delivery of GAA when there was a creatine transporter deficiency, we synthesized diacetyl guanidinoacetic acid ethyl ester (diacetyl-GAAE), a lipophilic derivative. In brain slices, 0.1 mM did not cause electrophysiological changes and improved tissue viability after blockage of the creatine transporter. However, diacetyl-GAAE did not increase creatine nor phosphocreatine in brain slices after blockage of the creatine transporter. We conclude that: (1) upon acute administration, GAA is neither epileptogenic nor neurotoxic; (2) Diacetyl-GAAE improves tissue viability after blockage of the creatine transporter but not through an increase in creatine or phosphocreatine. Diacetyl-GAAE might give rise to a GAA–phosphoGAA system that vicariates the missing creatine–phosphocreatine system. Our in vitro data show that GAA supplementation may be safe in the short term, and that a lipophilic GAA prodrug may be useful in creatine transporter deficiency.

## 1. Introduction

### 1.1. Creatine and Its Procurement by the Organism

**Figure 1:**
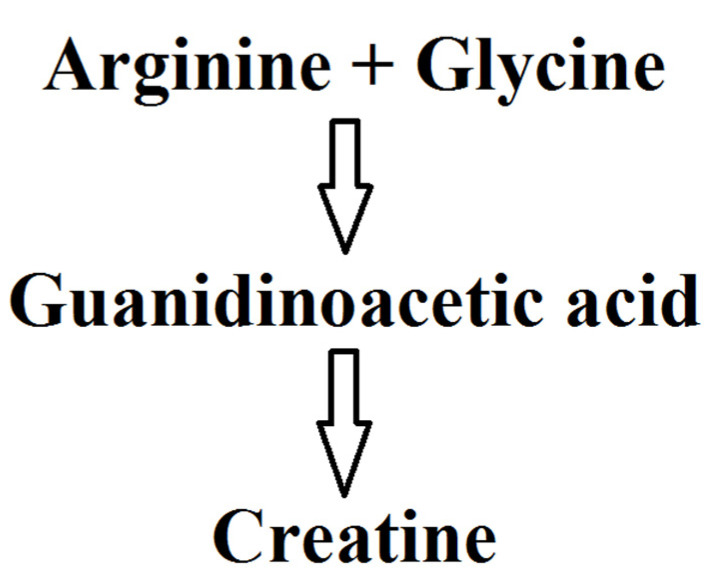
Endogenous synthesis of creatine, simplified scheme. See text for more details.

Creatine is of paramount importance in the energetic metabolism of excitable cells, where it serves both as a “shuttle” for rapidly transferring adenosine tri-phosphate (ATP) within the cytoplasm in normal functioning and as a buffer for ATP, preventing or delaying its exhaustion in pathological conditions [1,2,3,4]. Hereditary conditions where creatine is missing are attended by severe neurological symptoms including mental retardation, autism, speech disturbances, extrapyramidal movements, and epilepsy [5]. Creatine is both taken up from the diet and synthesized by the body, both sources equally contributing to creatine turnover in normal subjects [1]. Endogenous synthesis of creatine takes place in two steps [1], which Figure 1 illustrates in a simplified way.

Interestingly, this synthesis is accomplished in different organs, the first step (synthesis of guanidinoacetic acid from arginine and glycine) taking place in the kidney, the second one (synthesis of creatine from guanidinoacetic acid) in the liver [1]. Thus, guanidinoacetate is synthesized in the kidney, and then transported to the liver where it is converted to creatine, which is then transported to its destination organs [1]. The brain is capable of fully synthesizing its own creatine, the two steps again taking place in different cell types [6].

### 1.2. Use of Guanidinoacetate, the Creatine Precursor, as a Dietary Supplement

Although creatine is largely used as a dietary supplement in normal and pathological conditions [7,8], its precursor guanidinoacetate (Figure 1) has also been used experimentally to the same effect, in the belief that it would be converted into creatine once inside the cells [9]. It has even been reported that experimental supplementation with guanidinoacetic acid may increase tissue creatine more than supplementation with creatine itself [10].

However, supplementation with guanidinoacetic acid is considered with mixed feelings because it has been hypothesized that this compound may be toxic [6]. Specifically, it has been suggested that guanidinoacetic acid is responsible for the seizures and for the extrapyramidal movements that are often observed in a rare hereditary disease (guanidinoacetate-methyl transferase deficiency) where guanidinoacetate cannot be converted into creatine and accumulates in the interstitial space [11]. However, to the best of our knowledge, convincing experimental evidence of neurotoxicity by guanidinoacetic acid has never been provided, especially considering that the neurotoxic effects attributed to this compound were mostly observed at concentrations higher than those observed in clinical conditions [6], with the noteworthy exception of a study demonstrating that long term exposure to 10 micromol/L of guanidinoacetic acid (the concentration in the cerebrospinal fluid of GAMT-deficient patients) appears detrimental to the development of brain cells [12]. In fact, some of us [13] have even suggested that guanidinoacetic acid may be useful in GAMT deficiency at the muscular level because it is phosphorylated to phospho-guanidinoacetic acid and sets up a guanidinoacetate–phosphoguanidinoacetate system that may replace the creatine–phosphocreatine system when the latter is missing.

### 1.3. Possible Use of Guanidinoacetic Acid or of Its Prodrugs in Treating Creatine Transporter Deficiency

Finally, keeping in mind that guanidinoacetic acid is capable of increasing brain creatine in normal subjects [14], we hypothesized that it may be used to increase creatine content in patients affected by creatine transporter deficiency, a rare disease in which the creatine transporter is malfunctioning, preventing creatine uptake and synthesis by the brain [15]. Since, however, the same missing creatine transporter transports guanidinoacetic acid across the blood–brain barrier and from cell to cell [16], it is unlikely that it reaches the brain in creatine transporter deficiency. However, we hypothesized that if we modified the guanidinoacetic acid molecule and rendered it lipophilic it might cross the blood–brain barrier and cell plasma membranes independently of the transporter, in a fashion similar to creatine-derived, lipophilic molecules [17,18]. Moreover, it might represent a more physiologic way of replenishing brain creatine since it is believed that in the brain, creatine is normally synthesized by cells that uptake guanidinoacetic acid rather than being taken up as such [6].

### 1.4. Aims of This Paper

**Figure 2:**
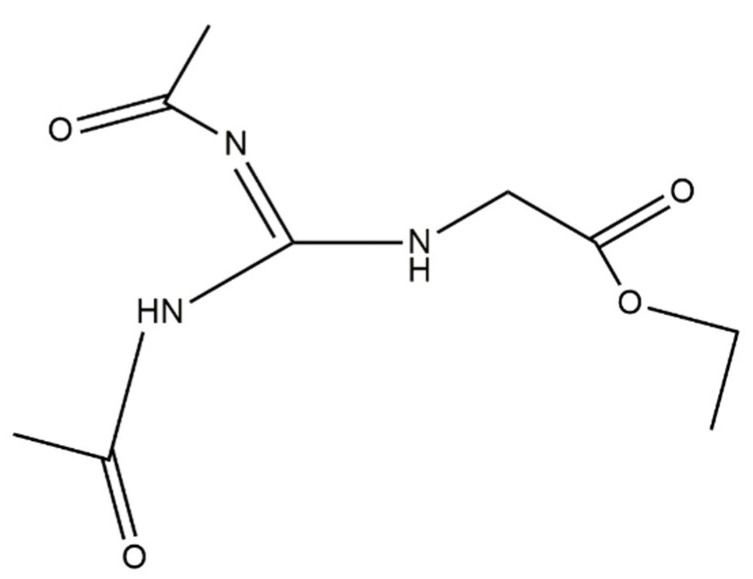
Chemical structure of *N*,*N*-diacetyl guanidine acetic ethyl ester (Diacetyl-GAAE).

Therefore, in the present paper we carried out experiments to investigate further whether or not guanidinoacetic acid is indeed toxic to brain cells. We then ideated and synthesized diacetyl-guanidine acetic acid ethyl ester (Diacetyl-GAAE, Figure 2), a lipophilic compound obtained by modifying guanidino acetic acid in a similar way to how we previously modified the creatine molecule [18].

Finally, we investigated if this modified molecule increased creatine content and afforded neuroprotection in brain slices where the creatine transporter was experimentally blocked.

## 2. Materials and Methods

### 2.1. Preparation of Hippocampal Slices

We used 28–40-day-old ICR CD1 albino Swiss mice, purchased from Envigo Italy (San Pietro al Natisone, Udine, Italy) and maintained at the animal facility of the Ospedale Policlinico San Martino. Approval for experiments was obtained from the Italian Ministry of Health (Ministero della Salute). Experiments were carried out in compliance with animal care requirements requested by Italian law (law D.L. 27.1.1992 n. 116, in agreement with the European Union directive 86/609/CEE). Formal approval to conduct the experiments was obtained from the Ethical Committee of the Animal Facility (OPBA) of the Ospedale Policlinico San Martino and can be provided upon request. All efforts were made to minimize the number of animals used and their suffering. Hippocampal slices were prepared in vitro as previously described (Adriano et al., 2011). To isolate the hippocampus, the animal was anesthetized with ethyl ether and decapitated. Subsequently, the left hippocampus was extracted and subdivided into transversal slices. The latter were 600 μm thick for electrophysiological experiments or 400 μm thick for biochemical experiments. The slices thus obtained were transferred into a serum-free solution of artificial cerebrospinal fluid constantly oxygenated by a mixture of 95% oxygen and 5% carbon dioxide, in this way, maintaining the pH around 7.35–7.4. Incubation temperature was 32 °C for electrophysiological experiments and 36 °C for biochemical experiments. The artificial cerebrospinal fluid (ACSF) is an aqueous solution made up of the following compounds: NaCl 130 mM; KCl 3.5 mM; NaH_2_PO_4_ 1.25 mM; NaHCO_3_ 24 mM; CaCl_2_ 31.2 mM; MgSO_4_ 21.2 mM; Glucose 410 mM (pH 7.35–7.4).

### 2.2. Inactivation of Creatine Transporter in Hippocampal Slices

The inactivation of the creatine transporter can be performed using either guanidinopropionic acid (GPA) or a chlorine-free incubation medium (Dai et al., 1999). We had already used both methods in our previous experiments [19,20]. Concentrations of GPA higher than 1 mM are, however, rather impractical, because in their presence no electrical activity could be elicited from the slices, probably due to a harmful effect on brain slices (preliminary results, not shown). Our previous data showed that incubation with GPA 1 mM reduced baseline creatine content by about 50% and reduced uptake of creatine by about the same measure [19]. In a similar way, chlorine-free incubation could not be used for electrophysiological experiments because removal of chloride obviously prevented neural excitation (not shown). By contrast, we prefer not to use GPA in experiments where creatine has to be measured with HPLC because GPA itself elutes in a region very close to creatine, thus interfering with its measurement (preliminary experiments, not shown). Thus, in the present experiments, we used GPA 1 mM to block CRT in experiments involving electrophysiological stimulation and recording, and chlorine-free incubation in experiments involving only biochemical measurements.

### 2.3. Study on the Effect of GPA on Slices Viability

Electrophysiological techniques were used on in vitro hippocampal slices to assess the harmful effects of GPA. Hippocampal slices from 4-week-old ICR male mice (CD-1) (Envigo farm) were obtained following the procedure reported elsewhere [21] and incubated for 3 h at 36 °C in one of the following incubation media:

- ACSF
- ACSF + GPA (0.5 mM)
- ACSF + GPA (1 mM)
- ACSF + GPA (1 mM) + Diacetyl-GAAE (0, 1 mM)

The slices were then transferred into an incubation and electrophysiological recording chamber (Fine Science Tools, Vancouver, Canada). Two microelectrodes were positioned in the hippocampal slices, a stimulating one in the Schaffer collaterals and a recording one in the CA1 cell body layer. Stimulation of the Schaffer collaterals caused the appearance at the recording site of a compound action potential (“Population Spike”, or PS), which is the extracellular hallmark of the firing of the CA1 neurons (Andersen et al., 1971). A smaller waveform (“Presynaptic Volley”, or PV) was also visible, which represents the firing of the presynaptic axons of the Schaffer collaterals. Slices were considered viable if a PS was present upon Schaffer collateral stimulation.

### 2.4. Tissue Processing for Biochemical Experiments

Four-hundred μm thick hippocampal slices were obtained and incubated at 36 °C for biochemical experiments in one of the following media:

- ACSF
- ACSF Cl-free
- ACSF + Creatine (2 mM)
- ACSF Cl-free + Creatine (2 mM)
- ACSF Cl-free + GAA (2 mM)
- ACSF Cl-free + Diacetyl-GAAE (0, 1 mM)

Slices were incubated for 180 min at 36 °C in the thermostatic bath, then washed with saline solution, instantly frozen in liquid nitrogen and immediately stored at −80 °C. Afterward, slices were homogenized in a solution of perchloric acid 0.3 M, to inactivate creatine kinase. The homogenate obtained was adjusted to pH 7 with potassium carbonate 3 M. Samples were centrifuged. Proteins in the precipitate were evaluated by bicinchoninic acid assay [22], using BCA Protein Assay Kit (Sigma-Aldrich); all the measures were performed using bovine serum albumin (BSA) as standard. The protein concentration was used to normalize the levels of different metabolites in the supernatant.

### 2.5. Synthesis of N-Diacetyl Guanidine Acetic Ethyl Ester (Diacetyl-GAAE)

**Figure 3:**
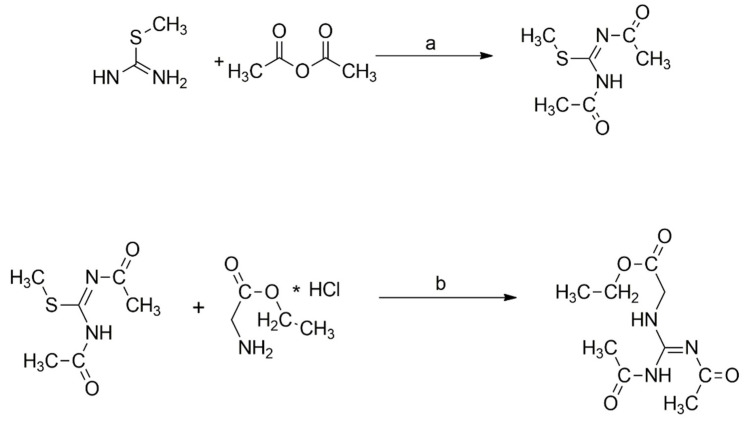
Synthesis of Diacetyl-GAAE. Reagents and conditions: (**a**) TEA, DCM, RT, 2 h; (**b**) TEA, HgCl2, THF, RT, 24 h. See text for more details.

This particular derivative cannot be synthesized starting from guanidine acid itself, but it must be obtained by using glycine ethyl ester. Figure 3 shows the various steps involved.

Briefly, the first step of the process required the use of two acetyl groups that were placed directly on the S-methylisothiourea to obtain a guanylating agent, which was protected on both its nitrogen atoms with acetyl groups. Therefore, this intermediate was conjugated with commercially available glycine ethyl, which was converted in good yield to diacetyl creatine ethyl ester derivative as final product. This protocol allowed its direct synthesis without sub-products. A more detailed description follows.

#### 2.5.1. Synthesis of Methyl *N*,*N*’-Diacetylcarbamimidothioate

To a solution of S-methylisothiourea (112 mg, 1.25 mmol, 1 eq) in anhydrous CH2Cl2 (0.5 mL), acetic anhydride (260 µL; 2,75 mmol, 2.2 eq) and triethylamine (TEA; 349 µL, 2.5 mmol, 2 eq) were added. The mixture was stirred for about two hours at room temperature until it changed color and turned dark. The reaction was then concentrated under reduced pressure. The resulting mixture was diluted with ethyl acetate (1 mL), washed with deionized water (3 × 1 mL), dried over Na_2_SO_4_, filtered and concentrated to provide a brown solid. The final product was then crystallized by absolute ethanol to provide the title compound (171 mg, yield 79%). The product was then used directly in the next step. The qualitative analysis of the product was finally confirmed by ESI-MS.

ESI-MS = 175.2 [M + H]+.

#### 2.5.2. Synthesis of (Z)-Ethyl 2-(2,3-Diacetylguanidino)acetate

Methyl *N*,*N*’-diacetylcarbamimidothioate (171 mg, 0.98 mmol) was dissolved in anhydrous tetrahydrofuran (THF) (2 mL); glycine ethyl ester hydrochloride (137 mg, 0.98 mmol, 1 eq), triethylamine (275 µL, 1.96 mmol, 2 eq), and HgCl2 (532 mg, 1.96 mmol, 2 eq) were added. The resulting mixture was stirred at room temperature upon completion of the reaction (controlled with HPLC-MS). The mixture was concentrated under reduced pressure and then resuspended in ethyl acetate (1 mL). The organic layer was washed with water (3 × 1 mL), dried over Na_2_SO_4_, filtered, and concentrated to provide a light brown semisolid. The final product was then crystallized by absolute ethanol to provide a solid. The final product was controlled in HPLC-MS to give the title compound (164 mg, 73%)

ESI-MS = 230.1 [M + H]+.

### 2.6. HPLC and Mass Spectrometry Analysis

The analytical instrument used was an Agilent 1260 high-performance liquid chromatograph (HPLC). The analytical HPLC column was a Waters Bondapack C18, 3.9 mm ID × 300 mm length. The analysis of the intermediates and the raw products was performed by liquid chromatography–electrospray mass spectrometry (HPLC-ESI-MS) using an Agilent 1100 series LC/MSD ion trap instrument. The separation was performed in gradient starting with 0% solvent B for 5 min, linearly increasing to 50% solvent B in 30 min and up to 100% B in 10 min. The solvents used were 0.1% formic acid in water (A) and 0.1% formic acid in acetonitrile (B). The molecular weight of the intermediates and of the final product was finally confirmed by electrospray mass spectrometry using an ion trap analyzer, which allowed performing MS and MS2 analysis.

### 2.7. Measurement of Tissue Content of Creatine

The quantitative analysis of metabolites was carried out, with minor modifications, according to a previously published paper [19], using high-performance liquid chromatography (Agilent 1260 system—Agilent Technologies, Palo Alto, CA, USA) equipped with a standard auto sampler. Briefly, separations were performed on an XTerra C18 column, 3.9 mm × 150 mm column with 5 µm particle size (Waters Corporation, Milford, MA, USA) in isocratic mode. The mobile phase was an aqueous solution of 40 mM sodium phosphate dibasic (Na_2_HPO_4_) containing 1 mM tetrabutylammonium hydrogen sulphate (TBAHS) (solvent A), while the mobile phase B was an aqueous solution of sodium phosphate monobasic (NaH_2_PO_4_) 40 mM, TBAHS 1 mM, at pH 6.8. All reagents were of analytical grade (Sigma, St. Louis, MO, USA). Flow rate was 0.8 mL/min and wavelengths were set to 220 nm. The column temperature was set to 30 °C. Injection volume was 10 μL both for standards and samples. Standard molecules were diluted in the mobile phase to reach the desired concentration, and the supernatants of the sample were diluted 1:1 in the mobile phase.

The calibration curves, performed in triplicate using three standard concentrations including the expected values for samples, were carried out before each set of samples and accuracy was verified daily. Sample concentrations were determined using sample peak areas. The accuracy was in a range from ±14 to ±17% at the tested levels and the relative standard deviation was in a range from 8% to 11%.

## 3. Results

### 3.1. Effects of Guanidinoacetic Acid

Patients affected by guanidinoacetate methyltransferase (GAMT) deficiency have a higher than normal concentration of guanidinoacetic acid (GAA) in their cerebrospinal fluid (CSF). Specifically, the range of their CSF content is 11.0–12.4 μmol/L, while that of normal controls is 0.068–0.114 [23] Thus, we tested the effects of a concentration that is relevant for GAMT-deficient patients, namely 11.5 μM.

**Figure 4:**
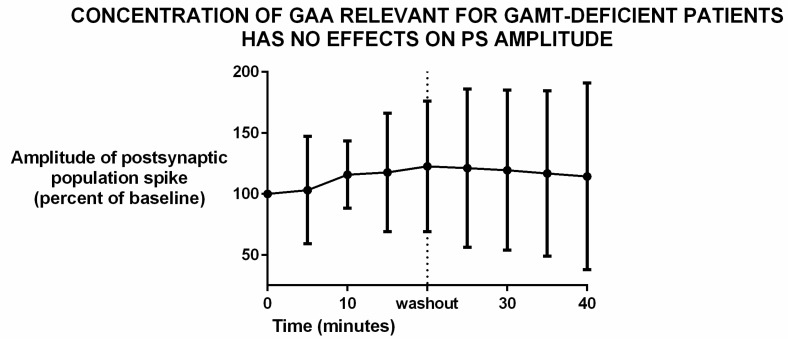
Incubation of brain slices with 11.5 μM guanidine acetic acid (GAA) did not change the amplitude of the postsynaptic evoked potential (“population spike”). Data represent mean and standard deviation. N = 12. Differences are statistically not significant (*p* = 0.98, analysis of variance). Slices were incubated with GAA for 20 min before washout. In most brain slices set up, including ours, this time is sufficient to allow exchange of chemicals to affect electrophysiological responses (personal data, not shown).

The incubation of brain slices with that rather high concentration of GAA did not have any effect on synaptic transmission. In fact, the amplitude of the extracellular compound action potential elicited by the presynaptic stimulation of the Schaffer collaterals did not change during the incubation (Figure 4 and Table S1).

**Figure 5:**
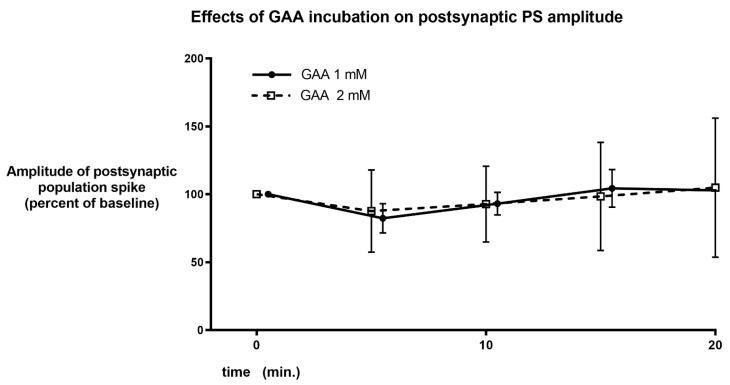
Same parameters and graph as in Figure 4, using higher concentrations of GAA. No change in PS amplitude is evident during GAA incubation.

**Figure 6:**
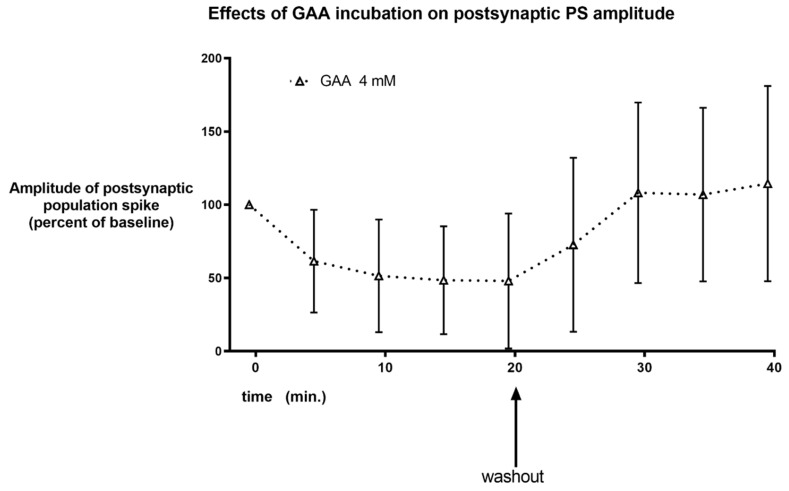
Same parameters and graph as in Figure 4 and Figure 5, using the concentration of 4 mM. A decrease in population spike amplitude is observed that was reversible upon washout and statistically significant (*p* = 0.0454, repeated measures analysis of variance).

Even using much higher concentrations of GAA did not cause neuronal hyperexcitability. Specifically, 1 and 2 mM did not cause any significant change in population spike amplitude (Figure 5 and Tables S2 and S3), while 4 mM GAA caused a not significant, reversible decrease in this postsynaptic potential (Figure 6 and Table S4).

**Figure 7:**
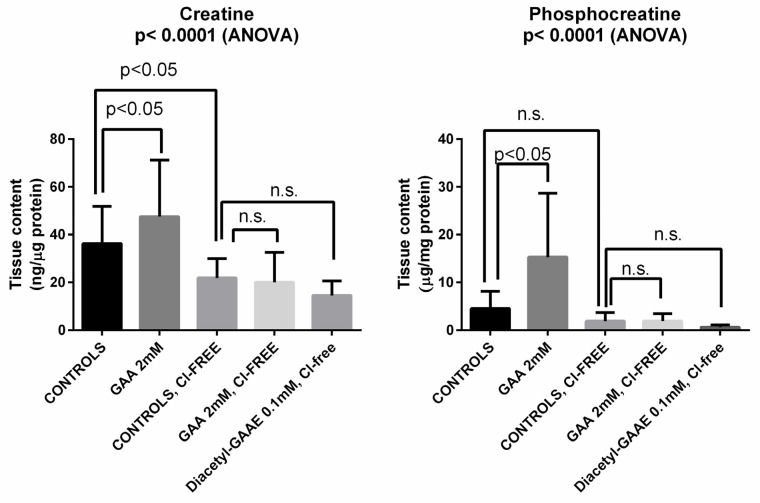
Incubation of brain slices with guanidine acetic acid (GAA) caused increase in brain slices’ content of creatine and phosphocreatine (compare first two columns in each graph). Such changes were statistically significant. Blocking the creatine transporter (which GAA also uses to enter cells [24]) by incubation with chloride-free medium caused a decrease in both compounds (compare first and third column in each graph), which was significant only for creatine. Furthermore, blocking the creatine transporter with Cl-free medium prevented the changes in creatine and phosphocreatine induced by GAA (compare third and fourth column in each graph). Diacetyl-GAAE, too, was unable to increase creatine and phosphocreatine after transporter block with Cl-free medium (compare third and fifth column in each graph). Statistics shown are for analysis of variance (ANOVA) or for Bonferroni’s multiple comparisons test; n.s. = not significant.

Incubation of brain slices with 2 mM GAA caused a significant increase in both creatine and phosphocreatine in the tissue. As expected, such an increase did not occur after blocking (obtained by removing Cl- from the ACSF) of the creatine transporter, which GAA also uses to enter cells [24]. We should note that incubation with Cl-free medium per se significantly reduced the brain slices’ content of creatine but did not significantly reduce that of phosphocreatine, similarly to what we have already reported [25]. Figure 7 and Tables S5 and S6 summarize these and other results (see below).

### 3.2. Diacetyl-Guanidinic Acid Ethyl Ester (Diacetyl-GAAE)

**Figure 8:**
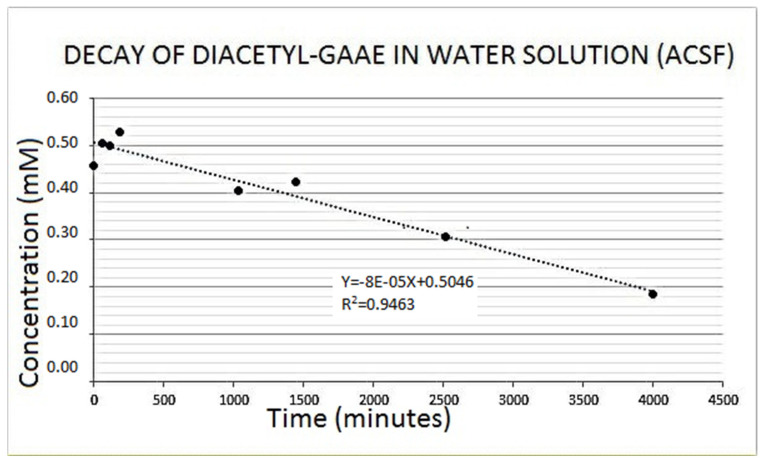
When dissolved in the aqueous artificial cerebrospinal fluid (ACSF) we used for brain slices’ incubation, Diacetyl-GAAE decayed linearly over time. Such a spontaneous decay was however very slow, and it did not occur in the period of our experiments. The latter lasted 3–4 h at most, a time when Diacetyl-GAAE concentration did not change. See text for additional details.

Diacetyl guanidinic acid ethyl ester (Diacetyl-GAAE) is a guanidine acetic acid derivative containing two acetyl groups on the guanidine nitrogens and an ethylic group on the carboxylic moiety (Figure 2; see also “Methods” section). Compared to guanidine acetic acid, the three added chains confer it as a very lipophilic character. We confirmed the purity of the compound using HPLC and mass spectrometry, as described in the methods. We checked the stability of the new synthetized derivative Diacetyl-GAAE in an aqueous solution. Figure 8 shows the spontaneous decay it undergoes in the watery ACSF we used for our experiments. Half time of decay was almost 3 days. Decay was negligible in the 3–4 h that were required for the experiments we described in this paper. Thus, degradation of Diacetyl-GAAE is very slow, and it certainly did not occur within the period of our experiments. Obviously, there is still the theoretical possibility that enzymes could degrade the compound in vivo, so further development of this or similar compounds shall include a pharmacokinetics study in vivo.

### 3.3. Effects of Diacetyl-GAAE

**Figure 9:**
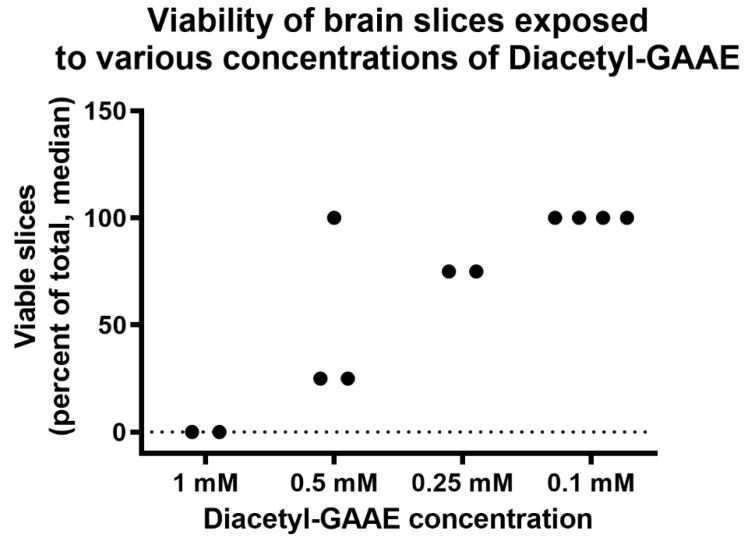
Percent of slices in each batch that showed synaptic transmission after incubation with various concentrations of Diacetyl-GAAE. *p* = 0.0089, analysis of variance.

We then tested the safety and toxicity of this compound. We used as an endpoint to this aim the amplitude of the postsynaptic compound action potential (“population spike”). Specifically, we investigated if Diacetyl-GAAE changed its amplitude. We, thus, tested the effects of Diacetyl-GAAE incubation for 3 h starting right after brain slices’ preparation. Under these conditions, Diacetyl-GAAE caused a dose-dependent loss of synaptic transmission. As Figure 9 and Table S7 show, only a concentration as low as 0.1 mM was associated with 100% viability of the slices under these conditions, while higher concentrations harmed synaptic transmission in a dose-dependent way.

Thus, we decided to use the concentration of 0.1 mM to test effectiveness. We emphasize that although loss of “population spike” may indicate structural damage of the slice [26], it might simply be a gauge of functional, reversible damage to the slice (for an example, see [27]). Nevertheless, this is an unwanted effect, which would discourage use of the compound at the harmful concentration.

**Figure 10:**
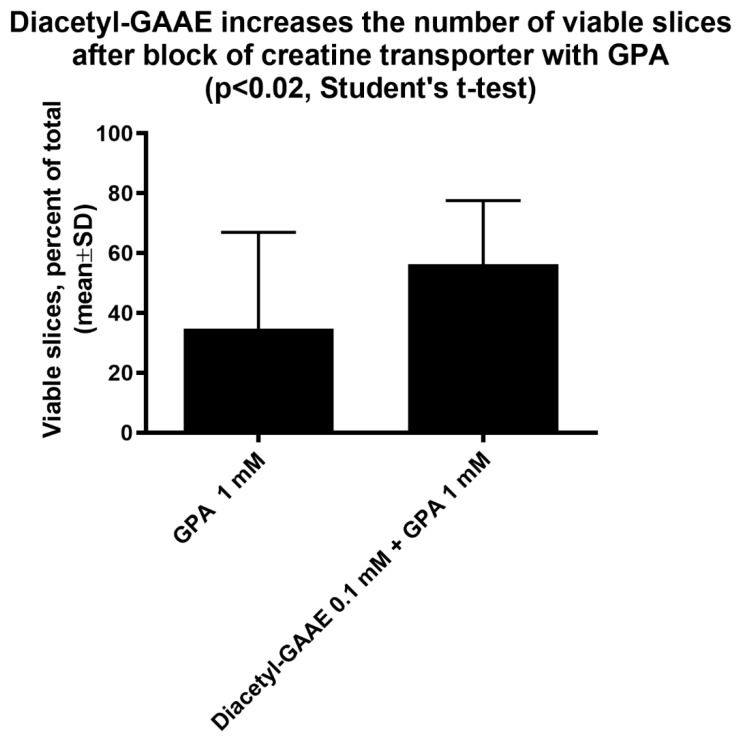
Slices were incubated with the creatine transporter block guanidine acetic acid (GPA). As expected, this treatment reduced drastically the percent of slices that in each batch maintained synaptic transmission (“viable” slices—note that control slices have a viability close to 100%, not shown). Co-incubation with Diacetyl-GAAE reduced this damage in a statistically significant way. Control slices were incubated with GPA 1 mM only, Diacetyl-GAA slices were incubated with GPA 1 mM and Diacetyl-GAA 0.1 mM. N = 18 and 20, respectively.

Treatment with Diacetyl-GAAE at this concentration improved the viability of the brain slices after a 3-h block of the creatine transporter with GPA (Figure 10 and Table S8). Viability was 35 ± 32% of the total in slices treated with GPA, 56 ± 21% in slices treated with both GPA and Diacetyl-GAAE (N = 18 and 20, respectively, *p* = 0.0359—Mann–Whitney test).

After blockage of the creatine transporter, Diacetyl-GAAE did not increase either creatine nor phosphocreatine (Figure 7, above). We shall report in the “Discussion” session (below) the possible explanation of this finding.

## 4. Discussion

It has been suggested that guanidino acetic acid (GAA), the precursor of creatine, may have an epileptogenic action in patients with guanidinoacetate methyl transferase (GAMT) deficiency [28], and strategies to decrease its levels in patients affected by GAMT deficiency are usually applied [29]. An inhibitory effect of GAA on brain Na+, K+-ATPase activity has been shown [30]. Nevertheless, to the best of our knowledge, no experimental evidence showing that GAA is indeed capable of causing epilepsy in normal subjects has been provided. Indeed, some of us have even reported that it has a role in improving the effects of GAMT deficiency in the muscle [13]. Moreover, GAA has been used as a dietary supplement in normal humans with no report of epileptic adverse events [9,10,14]. Thus, we investigated if, in brain slices, we could detect electrophysiological changes by GAA that might be suggestive of an epileptogenic action.

We were not able to demonstrate epileptic-like effects of GAA, either at a concentration that is relevant for GAMT-deficient patients (Figure 4), or at higher concentrations (Figure 5 and Figure 6). Furthermore, at the concentration of 2 Mm, the content of brain tissue creatine and phosphocreatine in normal slices increased (Figure 7). These results were not surprising since an increase in brain and muscle creatine after GAA treatment with no neurological side effects has been reported previously in humans [9,14].

We investigated the possible toxicity of GAA in the short term using the amplitude of the compound action potential (“population spike”) [31] as a gauge. We chose this electrophysiological parameter because (1) it is relevant to the claim that GAA may be epileptogenic, in fact if GAA is indeed acutely epileptogenic it should increase the synchrony of neuronal firing [32,33], thus increasing population spike amplitude [31]; (2) it is a sensitive gauge of tissue viability, thus, it is capable of detecting acute neural toxicity [26,34,35]. Therefore, the finding that in the short term GAA did not alter population spike amplitude until a very high concentration, strongly suggests that this compound is neither epileptogenic nor neurotoxic in the short term. This observation is consistent with the fact that no adverse effects have been reported after the use of GAA as a dietary supplement [9,10,14]. Issues remain open nevertheless. Specifically, one field where additional investigation may be warranted is the axonal hyper-sprouting that developing neurons show upon GAA treatment, which is followed by neuronal death [12]. Axonal hyper-sprouting has been claimed to be epileptogenic [36]. Therefore, while our data strongly suggest that GAA does not cause epilepsy by acutely altering synaptic transmission, different mechanisms of epileptogenesis are still theoretically possible. That no adverse events were reported upon GAA use as a dietary supplement in adults [9,10,14], suggests either that GAA is indeed not toxic, or that the adult brain may have a different susceptibility from the juvenile, developing brain. Further research should be carried out on these issues.

We think that the data on population spike amplitude we noted represent a significant support to our conclusion that GAA is not epileptogenic in the short term. Of course, additional electrophysiological measurements could be carried out, and further research should include them.

It has been suggested that in the brain, GAA is delivered to cells capable of synthesizing creatine [6,16]. Thus, delivering GAA to the brain may be a way to increase brain creatine in patients affected by creatine transporter deficiency. The latter is a rare hereditary condition, currently incurable, where lack of the creatine transporter prevents dietary creatine from reaching the brain and entering the cells where it is needed [15].

However, GAA did not increase creatine nor phosphocreatine content after blockage of the creatine transporter (Figure 7). This was expected, too, since GAA uses the same transport as creatine to enter the cells [24]. We then tested the effects of Diacetyl-GAAE, a lipophilic prodrug of GAA, in the same brain slices’ model, investigating if this lipophilic prodrug could counter the harmful effects of blocking the creatine transporter. Diacetyl-GAAE concentration of 0.1 mM did not affect the brain slices’ viability (Figure 9), we therefore considered this concentration to be safe and used it in our further experiments. It was indeed able to improve neuronal viability in a statistically significant way after blockage of the creatine transporter (Figure 10). However, it did not increase the creatine or phosphocreatine content of the brain slices; actually, it decreased both (Figure 7). We hypothesize that this discrepancy may be explained by the fact that once Diacetyl-GAAE is deacetylated and deprived of the ethyl ester moiety, it may be phosphorylated to phosphoGAA, setting up a GAA–phosphoGAA system that replaces the creatine–phosphocreatine system when the latter is absent [37]. Further research is needed to confirm this hypothesis. However, we previously found that cyclocreatine, a compound that also ameliorates the harmful effects of creatine transporter block in an animal model [38], may be phosphorylated as such in brain tissue, setting up a cyclocreatine–phosphocyclocreatine system that may be parallel to the creatine–phosphocreatine system [39]. Since cyclocreatine is effective in an in vivo model of creatine transporter deficiency [38], we hypothesize that Diacetyl-GAAE, too, may be effective in the same way.

As limitations of our study, we did not investigate the effects of GAA upon chronic administration, nor did we address possible differences in its effects between the developing and adult brain. Moreover, our data were obtained from in vitro experiments, so in vivo challenges should be investigated.

## 5. Conclusions

Summing up, we conclude that (1) GAA is neither epileptogenic nor neurotoxic, at least concerning synaptic alteration in the short term; (2) Diacetyl-GAAE improves tissue viability after blockage of the creatine transporter, but not through an increase in creatine or phosphocreatine. Diacetyl-GAAE might give rise to a GAA–phosphoGAA system that replaces the missing creatine–phosphocreatine system. Our data suggest that a lipophilic GAA prodrug may be a feasible way to treat creatine transporter deficiency and warrants further research on this topic.

## Data Availability

Data supporting reported results can be found as a supplementary tables, as quoted in the text.

## Supplementary Materials

The following supporting information can be downloaded at: https://www.mdpi.com/article/10.3390/brainsci12010085/s1, Figure S1: representative waveforms from one of our experiments (please note the following key to abbreviations: SA=stimulus artifact; PV=presynaptic volley; PS= population spike; EPSP=excitatory post synaptic potential, final part); Table S1: raw data of Figure 4; Table S2: raw data of Figure 5 (1 mM GAA); Table S3: raw data of Figure 5 (2 mM GAA); Table S4: raw data of Figure 6; Table S5: raw data of Figure 7 (creatine graph); Table S6: raw data of Figure 7 (phosphocreatine graph); Table S7: raw data of Figure 9; Table S8: raw data of Figure 10.

## References

[1] Wyss M., Kaddurah-Daouk R. (2000). Creatine and Creatinine Metabolism. Physiol. Rev..

[2] Greenhaff P.L. (2001). The Creatine-Phosphocreatine System: There’s More than One Song in Its Repertoire. J. Physiol..

[3] Adhihetty P.J., Beal M.F. (2008). Creatine and Its Potential Therapeutic Value for Targeting Cellular Energy Impairment in Neurodegenerative Diseases. Neuromol. Med..

[4] Wallimann T., Tokarska-Schlattner M., Schlattner U. (2011). The Creatine Kinase System and Pleiotropic Effects of Creatine. Amino Acids.

[5] Stockler-Ipsiroglu S., van Karnebeek C.D.M. (2014). Cerebral Creatine Deficiencies: A Group of Treatable Intellectual Developmental Disorders. Semin. Neurol..

[6] Hanna-El-Daher L., Braissant O. (2016). Creatine Synthesis and Exchanges between Brain Cells: What Can Be Learned from Human Creatine Deficiencies and Various Experimental Models?. Amino Acids.

[7] Kreider R.B., Kalman D.S., Antonio J., Ziegenfuss T.N., Wildman R., Collins R., Candow D.G., Kleiner S.M., Almada A.L., Lopez H.L. (2017). International Society of Sports Nutrition Position Stand: Safety and Efficacy of Creatine Supplementation in Exercise, Sport, and Medicine. J. Int. Soc. Sports Nutr..

[8] Balestrino M., Adriano E. (2019). Beyond Sports: Efficacy and Safety of Creatine Supplementation in Pathological or Paraphysiological Conditions of Brain and Muscle. Med. Res. Rev..

[9] Ostojic S.M., Drid P., Ostojic J. (2016). Guanidinoacetic Acid Increases Skeletal Muscle Creatine Stores in Healthy Men. Nutr. Burbank Los Angel. Cty. Calif.

[10] Ostojic S.M., Ostojic J., Drid P., Vranes M. (2016). Guanidinoacetic Acid versus Creatine for Improved Brain and Muscle Creatine Levels: A Superiority Pilot Trial in Healthy Men. Appl. Physiol. Nutr. Metab. Physiol. Appl. Nutr. Metab..

[11] Schütz P.W., Stöckler S., Gilman S. (2007). Creatine Deficiency Syndromes. Neurobiology of Disease.

[12] Hanna-El-Daher L., Béard E., Henry H., Tenenbaum L., Braissant O. (2015). Mild Guanidinoacetate Increase under Partial Guanidinoacetate Methyltransferase Deficiency Strongly Affects Brain Cell Development. Neurobiol. Dis..

[13] Balestrino M., Adriano E. (2020). Presence of Guanidinoacetate May Compensate Creatine Absence and Account for Less Statin-Induced Muscle Damage in GAMT-Deficient Compared to AGAT-Deficient Mice. Amino Acids.

[14] Ostojic S.M., Ostojic J., Drid P., Vranes M., Jovanov P. (2017). Dietary Guanidinoacetic Acid Increases Brain Creatine Levels in Healthy Men. Nutr. Burbank Los Angel. Cty. Calif.

[15] Van de Kamp J.M., Mancini G.M., Salomons G.S. (2014). X-Linked Creatine Transporter Deficiency: Clinical Aspects and Pathophysiology. J. Inherit. Metab. Dis..

[16] Braissant O. (2012). Creatine and Guanidinoacetate Transport at Blood-Brain and Blood-Cerebrospinal Fluid Barriers. J. Inherit. Metab. Dis..

[17] Trotier-Faurion A., Dézard S., Taran F., Valayannopoulos V., de Lonlay P., Mabondzo A. (2013). Synthesis and Biological Evaluation of New Creatine Fatty Esters Revealed Dodecyl Creatine Ester as a Promising Drug Candidate for the Treatment of the Creatine Transporter Deficiency. J. Med. Chem..

[18] Adriano E., Gulino M., Arkel M., Salis A., Damonte G., Liessi N., Millo E., Garbati P., Balestrino M. (2018). Di-Acetyl Creatine Ethyl Ester, a New Creatine Derivative for the Possible Treatment of Creatine Transporter Deficiency. Neurosci. Lett..

[19] Lunardi G., Parodi A., Perasso L., Pohvozcheva A.V., Scarrone S., Adriano E., Florio T., Gandolfo C., Cupello A., Burov S.V. (2006). The Creatine Transporter Mediates the Uptake of Creatine by Brain Tissue, but Not the Uptake of Two Creatine-Derived Compounds. Neuroscience.

[20] Adriano E., Garbati P., Salis A., Damonte G., Millo E., Balestrino M. (2017). Creatine Salts Provide Neuroprotection Even after Partial Impairment of the Creatine Transporter. Neuroscience.

[21] Whittingham T.S., Lipton P. (1981). Cerebral Synaptic Transmission During Anoxia Is Protected by Creatine. J. Neurochem..

[22] Smith P.K., Krohn R.I., Hermanson G.T., Mallia A.K., Gartner F.H., Provenzano M.D., Fujimoto E.K., Goeke N.M., Olson B.J., Klenk D.C. (1985). Measurement of Protein Using Bicinchoninic Acid. Anal. Biochem..

[23] Caldeira Araújo H., Smit W., Verhoeven N.M., Salomons G.S., Silva S., Vasconcelos R., Tomás H., Tavares de Almeida I., Jakobs C., Duran M. (2005). Guanidinoacetate Methyltransferase Deficiency Identified in Adults and a Child with Mental Retardation. Am. J. Med. Genet. A.

[24] Tachikawa M., Hosoya K.-I. (2011). Transport Characteristics of Guanidino Compounds at the Blood-Brain Barrier and Blood-Cerebrospinal Fluid Barrier: Relevance to Neural Disorders. Fluids Barriers CNS.

[25] Garbati P., Adriano E., Salis A., Ravera S., Damonte G., Millo E., Balestrino M. (2014). Effects of Amide Creatine Derivatives in Brain Hippocampal Slices, and Their Possible Usefulness for Curing Creatine Transporter Deficiency. Neurochem. Res..

[26] Melani R., Rebaudo R., Noraberg J., Zimmer J., Balestrino M. (2005). Changes in Extracellular Action Potential Detect Kainic Acid and Trimethyltin Toxicity in Hippocampal Slice Preparations Earlier than Do MAP2 Density Measurements. Altern. Lab. Anim. ATLA.

[27] Furling D., Ghribi O., Lahsaini A., Mirault M.E., Massicotte G. (2000). Impairment of Synaptic Transmission by Transient Hypoxia in Hippocampal Slices: Improved Recovery in Glutathione Peroxidase Transgenic Mice. Proc. Natl. Acad. Sci. USA.

[28] Schulze A., Ebinger F., Rating D., Mayatepek E. (2001). Improving Treatment of Guanidinoacetate Methyltransferase Deficiency: Reduction of Guanidinoacetic Acid in Body Fluids by Arginine Restriction and Ornithine Supplementation. Mol. Genet. Metab..

[29] Viau K.S., Ernst S.L., Pasquali M., Botto L.D., Hedlund G., Longo N. (2013). Evidence-Based Treatment of Guanidinoacetate Methyltransferase (GAMT) Deficiency. Mol. Genet. Metab..

[30] Zugno A.I., Franzon R., Chiarani F., Bavaresco C.S., Wannmacher C.M.D., Wajner M., Wyse A.T.S. (2004). Evaluation of the Mechanism Underlying the Inhibitory Effect of Guanidinoacetate on Brain Na^+^, K^+^-ATPase Activity. Int. J. Dev. Neurosci. Off. J. Int. Soc. Dev. Neurosci..

[31] Andersen P., Bliss T.V.P., Skrede K.K. (1971). Unit Analysis of Hippocampal Population Spikes. Exp. Brain Res..

[32] Margineanu D.G. (2010). Epileptic Hypersynchrony Revisited. Neuroreport.

[33] Westbrook G., Kandel E.R., Jessel T.M., Schwartz J.H. (1991). Seizures and Epilepsy. Principles of Neural Science.

[34] Köhling R., Melani R., Koch U., Speckmann E.-J., Koudelka-Hep M., Thiébaud P., Balestrino M. (2005). Detection of Electrophysiological Indicators of Neurotoxicity in Human and Rat Brain Slices by a Three-Dimensional Microelectrode Array. Altern. Lab. Anim. ATLA.

[35] Van Vliet E., Stoppini L., Balestrino M., Eskes C., Griesinger C., Sobanski T., Whelan M., Hartung T., Coecke S. (2007). Electrophysiological Recording of Re-Aggregating Brain Cell Cultures on Multi-Electrode Arrays to Detect Acute Neurotoxic Effects. Neurotoxicology.

[36] Bausch S.B. (2005). Axonal Sprouting of GABAergic Interneurons in Temporal Lobe Epilepsy. Epilepsy Behav. EB.

[37] Kan H.E., Renema W.K.J., Isbrandt D., Heerschap A. (2004). Phosphorylated Guanidinoacetate Partly Compensates for the Lack of Phosphocreatine in Skeletal Muscle of Mice Lacking Guanidinoacetate Methyltransferase. J. Physiol..

[38] Kurosawa Y., Degrauw T.J., Lindquist D.M., Blanco V.M., Pyne-Geithman G.J., Daikoku T., Chambers J.B., Benoit S.C., Clark J.F. (2012). Cyclocreatine Treatment Improves Cognition in Mice with Creatine Transporter Deficiency. J. Clin. Investig..

[39] Enrico A., Patrizia G., Luisa P., Alessandro P., Gianluigi L., Carlo G., Maurizio B. (2013). Electrophysiology and Biochemical Analysis of Cyclocreatine Uptake and Effect in Hippocampal Slices. J. Integr. Neurosci..

