# Supplementary material for: Effects of Delivering Guanidinoacetic Acid or Its Prodrug to the Neural Tissue: Possible Relevance for Creatine Transporter Deficiency
Source: Brain Sci. 2022 Jan 7;12(1):85. doi: 10.3390/brainsci12010085 (PMC8773658; doi:10.3390/brainsci12010085)
Supplement: Supplementary file 1 [file brainsci-12-00085-s001.zip › Table S1.pdf]

		Time from infusion start (minutes)								
		0	5	10	15	20	25	30	35	40
Amplitude of population spike (percent of baseline)	Subject 1	100	104	107	100	100	107	118	100	89
	Subject 2	100	111	123	140	217	238	168	162	138
	Subject 3	100	158	138	159	177	184	206	216	230
	Subject 4	100	9	108	115	109	71	43	26	34
	Subject 5	100	132	127	116	94	64	61	65	47
	Subject 6	100	144	152	158	168	184	208	224	232
	Subject 7	100	111	111	195	116	137	142	137	132
	Subject 8	100	121	128	158	165	174	188	165	216
	Subject 9	100	108	112	35	25	13	9	13	19
	Subject 10	100	24	42	47	54	59	66	69	71
	Subject 11	100	119	105	70	119	104	111	99	109
	Subject 12	100	97	138	121	128	119	114	124	55
	**Median**	**100**	**111**	**117**	**118**	**117**	**113**	**116**	**112**	**99**
	**Mean**	**100**	**103**	**116**	**118**	**123**	**121**	**119**	**117**	**114**
	**Std. Deviation**	**0,0**	**44**	**28**	**49**	**54**	**65**	**66**	**68**	**77**

Supplemental Table S1 – Amplitude of postsynaptic population spike after infusion with 11.5 µM of guanidinoacetic acid. Differences within columns are statistically not significant (p=0.57, Repeated measures ANOVA).
