# Supplementary material for: Effects of Delivering Guanidinoacetic Acid or Its Prodrug to the Neural Tissue: Possible Relevance for Creatine Transporter Deficiency
Source: Brain Sci. 2022 Jan 7;12(1):85. doi: 10.3390/brainsci12010085 (PMC8773658; doi:10.3390/brainsci12010085)
Supplement: Supplementary file 1 [file brainsci-12-00085-s001.zip › Table S2.pdf]

		Time from infusion start (minutes)				
		0	5	10	15	20
Amplitude of population spike (percent of baseline)	Subject 1	0	5	10	15	20
	Subject 2	100,000	78,72340	69,14893	67,55319	56,38298
	Subject 3	100,000	74,35897	61,53846	46,15385	41,02564
	Subject 4	100,000	100,73530	101,47060	116,17650	125,00000
	Subject 5	100,000	104,44440	115,55560	104,44440	135,55560
	Subject 6	100,000	23,07692	84,61539	146,15380	130,76920
	Subject 7	100,000	96,15385	101,92310	107,69230	110,57690
	Subject 8	100,000	98,91304	117,39130	142,39130	119,56520
	**Median**	**100**	**96**	**101**	**108**	**120**
	**Mean**	**100**	**82**	**93**	**104**	**103**
	**Std. Deviation**	**0,0**	**29**	**22**	**37**	**38**

Supplemental Table S2 – Amplitude of postsynaptic population spike after infusion with 1mM of guanidinoacetic acid. Differences within columns are statistically not significant (p=0.37, Repeated measures ANOVA).
