# Supplementary material for: Effects of Delivering Guanidinoacetic Acid or Its Prodrug to the Neural Tissue: Possible Relevance for Creatine Transporter Deficiency
Source: Brain Sci. 2022 Jan 7;12(1):85. doi: 10.3390/brainsci12010085 (PMC8773658; doi:10.3390/brainsci12010085)
Supplement: Supplementary file 1 [file brainsci-12-00085-s001.zip › Table S3.pdf]

		Time from infusion start (minutes)				
		0	5	10	15	20
Amplitude of population spike (percent of baseline)	Subject 1	100,	93,939390	96,96970	112,12120	127,272700
	Subject 2	100,	83,333340	62,50000	70,83334	54,166670
	Subject 3	100,	67,741940	53,22581	25,80645	25,806450
	Subject 4	100,	95,238100	100,00000	104,76190	95,238100
	Subject 5	100,	34,567900	90,12346	104,93830	112,345700
	Subject 6	100,	128,409100	134,46970	150,75760	171,212100
	Subject 7	100,	110,000000	112,00000	120,00000	148,000000
	**Median**	**100**	**94**	**97**	**105**	**112**
	**Mean**	**100**	**88**	**93**	**98**	**105**
	**Std. Deviation**	**0,0**	**30**	**28**	**40**	**51**

Supplemental Table S3 – Amplitude of postsynaptic population spike after infusion with 2mM of guanidinoacetic acid. Differences within columns are statistically not significant (p=0.54, Repeated measures ANOVA).
