# Supplementary material for: Effects of Delivering Guanidinoacetic Acid or Its Prodrug to the Neural Tissue: Possible Relevance for Creatine Transporter Deficiency
Source: Brain Sci. 2022 Jan 7;12(1):85. doi: 10.3390/brainsci12010085 (PMC8773658; doi:10.3390/brainsci12010085)
Supplement: Supplementary file 1 [file brainsci-12-00085-s001.zip › Table S4.pdf]

		Time from infusion start (minutes)								
		0	5	10	15	20	25	30	35	40
Amplitude of population spike (percent of baseline)	Subject 1	100,	81,05264	51,57895	41,05263	36,842110	108,42110	168,42110	180,00000	201,05260
	Subject 2	100,	66,53696	59,14397	53,69650	42,412450	73,54086	76,65369	68,87160	70,03891
	Subject 3	100,	37,93103	24,13793	27,58621	24,137930	13,79310	51,72414	51,72414	51,72414
	Subject 4	100,	16,27907	11,62791	11,62791	9,302325	16,27907	62,79070	72,09303	79,06977
	Subject 5	100,	105,40540	110,81080	108,10810	127,027000	151,35130	181,08110	162,16220	170,27030
	**Median**	**100**	**67**	**52**	**41**	**37**	**74**	**77**	**72**	**79**
	**Mean**	**100**	**61**	**51**	**48**	**48**	**73**	**108**	**107**	**114**
	**Std. Deviation**	**0,0**	**35**	**38**	**37**	**46**	**59**	**62**	**59**	**67**

Supplemental Table S4 – Amplitude of postsynaptic population spike after infusion with 4mM of guanidinoacetic acid. Differences within columns are statistically significant (p=0.04, Repeated measures ANOVA).
