# Supplementary material for: Effects of Delivering Guanidinoacetic Acid or Its Prodrug to the Neural Tissue: Possible Relevance for Creatine Transporter Deficiency
Source: Brain Sci. 2022 Jan 7;12(1):85. doi: 10.3390/brainsci12010085 (PMC8773658; doi:10.3390/brainsci12010085)
Supplement: Supplementary file 1 [file brainsci-12-00085-s001.zip › Table S5.pdf]

TISSUE CREATINE CONTENT AFTER INCUBATION IN VARIOUS EXPERIMENTAL CONDITIONS

	CONTROLS	CONTROLS, Cl-FREE	GAA 2mM	GAA 2mM, Cl-FREE	Diacetyl-GAAE 0.1mM, Cl-free
	35,310	19,833	29,500	28,523	22,420
	14,080	12,226	82,470	32,614	25,380
	10,350	14,898	35,170	25,323	0,000
	4,510	24,691	33,340	15,827	19,430
	20,780	47,486	36,490	3,390	12,220
	26,000	13,668	19,780	16,618	10,260
	40,540	13,542	43,360	4,555	12,560
	40,020	22,699	55,630	6,441	12,790
	28,340	17,596	30,410	38,331	17,864
	46,920	13,406	30,750	29,156	12,354
	46,340	19,090	46,987		19,191
	44,030	20,920	39,883		10,674
	57,920	23,000	38,277		18,437
	35,480	21,810	105,488		11,112
	34,170	34,110	88,219		13,437
	15,910	19,940	11,732		
	61,720	25,410	93,949		
	24,760	21,700	21,342		
	49,590	17,720	62,901		
	38,600	24,570	28,114		
	45,290	22,260	58,470		
	44,310	24,170	50,793		
	57,630	19,130	37,584		
	43,960	15,300	39,472		
	54,230	38,580	59,997		
	48,900		72,185		
	47,290		59,088		
	34,710		50,664		
	10,280		19,516		
	46,160		11,793		
	43,710		71,736		
	10,060		56,794		
**MEDIAN**	**40**	**21**	**42**	**21**	**13**
**MEAN**	**36**	**22**	**48**	**20**	**15**
**ST. DEV.**	**16**	**8,1**	**24**	**13**	**6,1**

Supplemental Table S5 – Tissue creatine content (ng/μg protein) in the various experimental conditions.
