# Supplementary material for: Effects of Delivering Guanidinoacetic Acid or Its Prodrug to the Neural Tissue: Possible Relevance for Creatine Transporter Deficiency
Source: Brain Sci. 2022 Jan 7;12(1):85. doi: 10.3390/brainsci12010085 (PMC8773658; doi:10.3390/brainsci12010085)
Supplement: Supplementary file 1 [file brainsci-12-00085-s001.zip › Table S6.pdf]

TISSUE PHOSPHOCREATINE CONTENT AFTER INCUBATION IN VARIOUS EXPERIMENTAL CONDITIONS

	CONTROLS	CONTROLS, Cl-FREE	GAA 2mM	GAA 2mM, Cl-FREE	Diacetyl-GAAE 0.1mM, Cl-free
	13,327	1,190	13,590	3,377	0,764
	10,888	0,236	12,190	4,221	1,116
	1,508	1,054	15,730	4,142	0,000
	0,564	3,175	23,830	1,874	0,821
	2,901	7,212	15,880	0,190	0,431
	2,768	0,473	7,710	1,553	0,000
	3,547	1,140	17,920	0,395	0,619
	4,025	0,471	26,300	0,829	1,006
	6,127	2,672	16,270	0,717	0,777
	6,068	0,664	13,930	2,306	1,222
	4,060	1,650	5,882		0,000
	3,950	0,980	20,868		0,613
	4,490	1,940	23,460		1,751
	2,430	1,850	49,517		0,000
	2,520	2,120	31,273		0,517
	0,970	1,590	5,847		
	5,100	6,780	41,742		
	2,170	1,950	10,530		
	3,790	0,770	25,496		
	1,870	1,850	13,597		
	3,600	0,680	27,995		
	4,600	1,270	44,970		
	16,750	3,630	7,218		
	5,330	0,000	0,000		
	3,320	2,920	2,795		
	3,000		3,005		
	8,960		3,179		
	2,160		2,079		
	0,750		0,537		
	4,910		0,619		
	7,720		3,433		
	0,960		2,232		
**MEDIAN**	**3,7**	**1,6**	**14**	**1,7**	**0,62**
**MEAN**	**4,5**	**1,9**	**15**	**2,0**	**0,64**
**ST. DEV.**	**3,6**	**1,8**	**13**	**1,5**	**0,51**

Supplemental Table S6 – Tissue phosphocreatine content (ng/µg protein) in the various experimental conditions.
