# Supplementary material for: Effects of Delivering Guanidinoacetic Acid or Its Prodrug to the Neural Tissue: Possible Relevance for Creatine Transporter Deficiency
Source: Brain Sci. 2022 Jan 7;12(1):85. doi: 10.3390/brainsci12010085 (PMC8773658; doi:10.3390/brainsci12010085)
Supplement: Supplementary file 1 [file brainsci-12-00085-s001.zip › Table S7.pdf]

	Concentration of Diacetyl-GAAE			
	1mM	0.5mM	0.25mM	0.1mM
Viable slices in each experiment (percent of total)	0,	100,	75,	100,
	0,	25,	75,	100,
		25,		100,
				100,

Supplemental Table S7 – Effect of various concentrations of Diacetyl-GAAE on slices viability. Data in each cell are the percentage of viable slices in a single experiment with the specified concentration of Diacetyl-GAAE.
