# Supplementary material for: Effects of Delivering Guanidinoacetic Acid or Its Prodrug to the Neural Tissue: Possible Relevance for Creatine Transporter Deficiency
Source: Brain Sci. 2022 Jan 7;12(1):85. doi: 10.3390/brainsci12010085 (PMC8773658; doi:10.3390/brainsci12010085)
Supplement: Supplementary file 1 [file brainsci-12-00085-s001.zip › Table S8.pdf]

	GPA1mM	Diacetyl-GAAE 0.1 mM + GPA 1mM
	0,	25,
	75,	75,
	75,	100,
	0,	75,
	75,	75,
	25,	25,
	0,	50,
	0,	50,
	25,	25,
	25,	25,
	75,	50,
	75,	75,
	0,	50,
	25,	50,
	50,	50,
	25,	75,
	0,	50,
	75,	75,
		50,
		75,
**MEDIAN**	**25**	**50**
**MEAN**	**35**	**56**
**ST. DEV.**	**32**	**21**

Supplemental Table S8 – Effect of Diacetyl-GAAE on slices viability after block of creatine transporter with 1mM guanidinopropionic acid (GPA). Data in each cell are the percentage of viable slices in a single experiment with the specified treatment.
