# Supplementary figures and images for: Effects of Delivering Guanidinoacetic Acid or Its Prodrug to the Neural Tissue: Possible Relevance for Creatine Transporter Deficiency
Source: Brain Sci. 2022 Jan 7;12(1):85. doi: 10.3390/brainsci12010085 (PMC8773658; doi:10.3390/brainsci12010085)

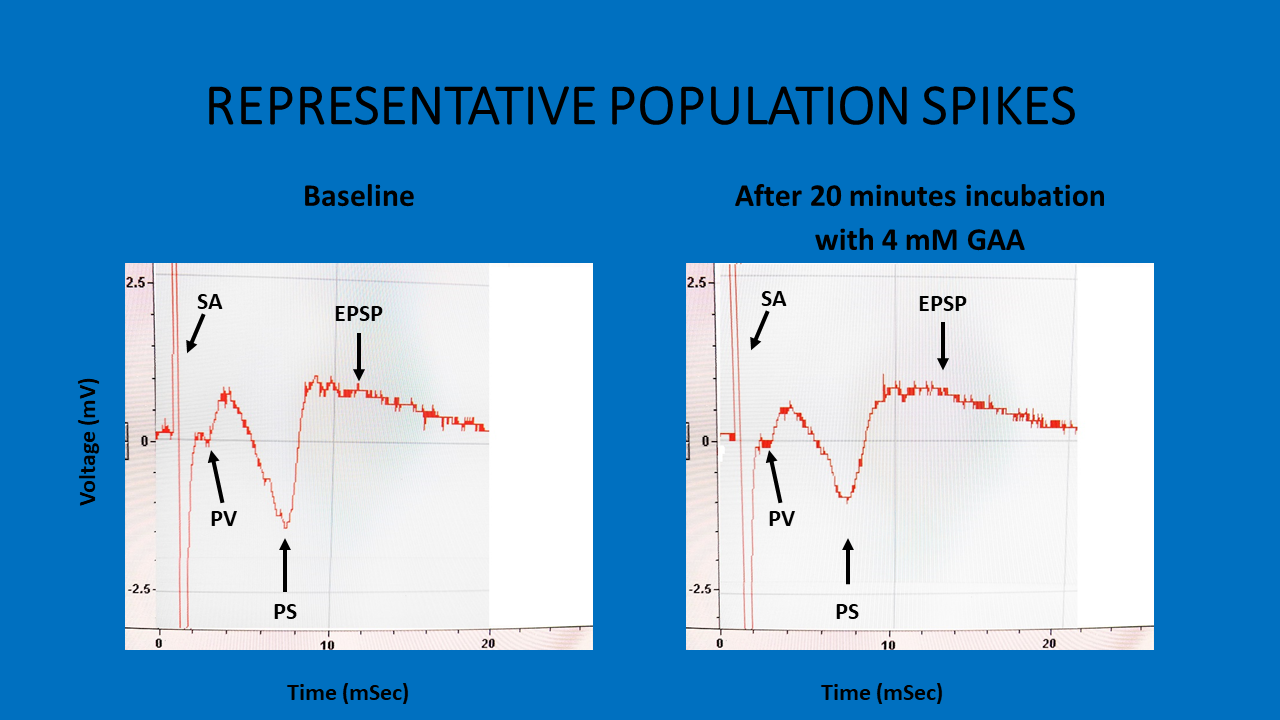

Supplement: Supplementary file 1 [file brainsci-12-00085-s001.zip › Figure S1.tif]
